# Supplementary material for: Venetoclax with Hypomethylating Agents in Newly Diagnosed Acute Myeloid Leukemia: A Systematic Review and Meta-Analysis of Survival Data from Real-World Studies
Source: Cancers (Basel). 2023 Sep 18;15(18):4618. doi: 10.3390/cancers15184618 (PMC10526951; doi:10.3390/cancers15184618)
Supplement: Supplementary file 1 [file cancers-15-04618-s001.zip › cancers-2581443-supplementary.pdf]

Supplementary Materials

Table S1. Baseline clinical characteristics of the patients.

Study	ELN intermediate - %	Normal karyotype - %	Bone marrow blasts – % (range)	Hemoglobin (g/dl) – mean (range)	White blood cells (10^3/µl) – mean (range)	Platelets (10^3/µl) – mean (range)
Aiba, 2023 [28]	nr	nr	nr	nr	nr	nr
Apel, 2021 [29]	42	35	40	9	5	73
Begna, 2021 [30]	nr	nr	nr	nr	nr	nr
Cherry, 2021 [31]	17	nr	50 (10-97)	nr	nr	nr
De Bellis, 2022 [21]	55	38	55 (20-100)	9 (4-15)	5 (<1-109)	39 (7-351)
Feld, 2021 [32]	43	nr	25	9	5	58
Fleishmann, 2022 [33]	30	32	nr	nr	12 (<1-317)	nr
Garciaz, 2022 [34]	nr	nr	36 (7-88)	nr	4 (<1-63)	90 (3-365)
Kwang, 2022 [35]	61	nr	51	nr	nr	nr
Matthews, 2022 [26]	nr	nr	nr	nr	nr	nr
Mirgh, 2021 [36]	63	42	nr	8 (5-11)	nr	nr
Morsia, 2020 [37]	27	41	35 (20-95)	9 (5-14)	4 (1-97)	73 (10-444)
Mustafa Ali, 2022 [38]	82	nr	nr	nr	nr	nr
Salhotra, 2021 [39]	20	nr	41 (20-90)	nr	15 (<1-127)	110 (6-771)
Shah, 2022 [40]	nr	nr	27	9	3	87
Todisco, 2023 [24]	54	nr	40	9	6	43
Vachhani, 2022 [27]	22	nr	nr	nr	nr	nr
Venugopal, 2021 [41]	nr	3	33 (8-88)	nr	3 (1-73)	34 (5-136)
Winters, 2019 [42]	10	nr	55	nr	3	nr
DiNardo, 2020 [14]	nr	44	<30% 30%<br>30%-50% 21%<br>50% 49%	With Anemia 31%	With Neutropenia 72%	With Thrombocytopenia 51%

ELN: European LeukemiaNet risk classification; nr not reported for the considered subgroup.

Table S2. Reported Adverse Events (AE).

Study	N. patients	Neutropenia grade 3/4 - %	Thrombocytopenia - %	Febrile neutropenia - %	TLS - %	Discontinuation for AE - %
Aiba, 2023 [28]	13	nr	nr	80	nr	nr
Apel, 2021 [29]	133	88	75	62	12	8
Begna, 2021 [30]	28	nr	nr	nr	nr	nr
Cherry, 2021 [31]	143	nr	nr	nr	nr	nr
De Bellis, 2022 [21]	51	29	14	13	0	55
Feld, 2021 [32]	72	72	nr	47	6	35
Fleishmann, 2022 [33]	17	45	15	nr	12	29
Garciaz, 2022 [34]	39	nr	nr	nr	nr	nr
Kwang, 2022 [35]	74	nr	nr	nr	1	7
Matthews, 2022 [26]	129	31	30	54	nr	nr
Mirgh, 2021 [36]	24	70	38	33	1.8	66
Morsia, 2020 [37]	44	nr	nr	nr	nr	30
Mustafa Ali, 2022 [38]	51	nr	nr	nr	nr	nr
Salhotra, 2021 [39]	30	nr	nr	nr	nr	nr
Shah, 2022 [40]	48	nr	nr	nr	nr	nr
Todisco, 2023 [24]	43	37	21	19	0	11
Vachhani, 2022 [27]	169	26	nr	nr	nr	12
Venugopal, 2021 [41]	58	nr	nr	nr	nr	nr
Winters, 2019 [42]	30	93	90	47	nr	nr
DiNardo, 2020 [14]	286	42	45	42	1	24

nr: not reported for the considered subgroup; TLS: tumor lysis syndrome.

Table S3. *p*-values of the log-rank test for the pairwise comparisons between single studies and DiNardo et al.

Study	p-value
Aiba, 2023 [28]	0.5651
Apel, 2021 [29]	0.0136
Begna, 2021 [30]	0.1734
Cherry, 2021 [31]	0.5787
De Bellis, 2022 [21]	0.5207
Feld, 2021 [32]	0.0873
Fleishmann, 2022 [33]	0.6061
Garciaz, 2022 [34]	0.0585
Kwang, 2022 [35]	0.6534
Matthews, 2022 [26]	0.0259
Mirgh, 2021 [36]	0.4590
Morsia, 2020 [37]	0.1707
Mustafa Ali, 2022 [38]	0.0013
Salhotra, 2021 [39]	0.3333
Shah, 2022 [40]	0.1420
Todisco, 2023 [24]	0.0249
Vachhani, 2022 [27]	<0.0001
Venugopal, 2021 [41]	<0.0001
Winters, 2019 [42]	0.0001

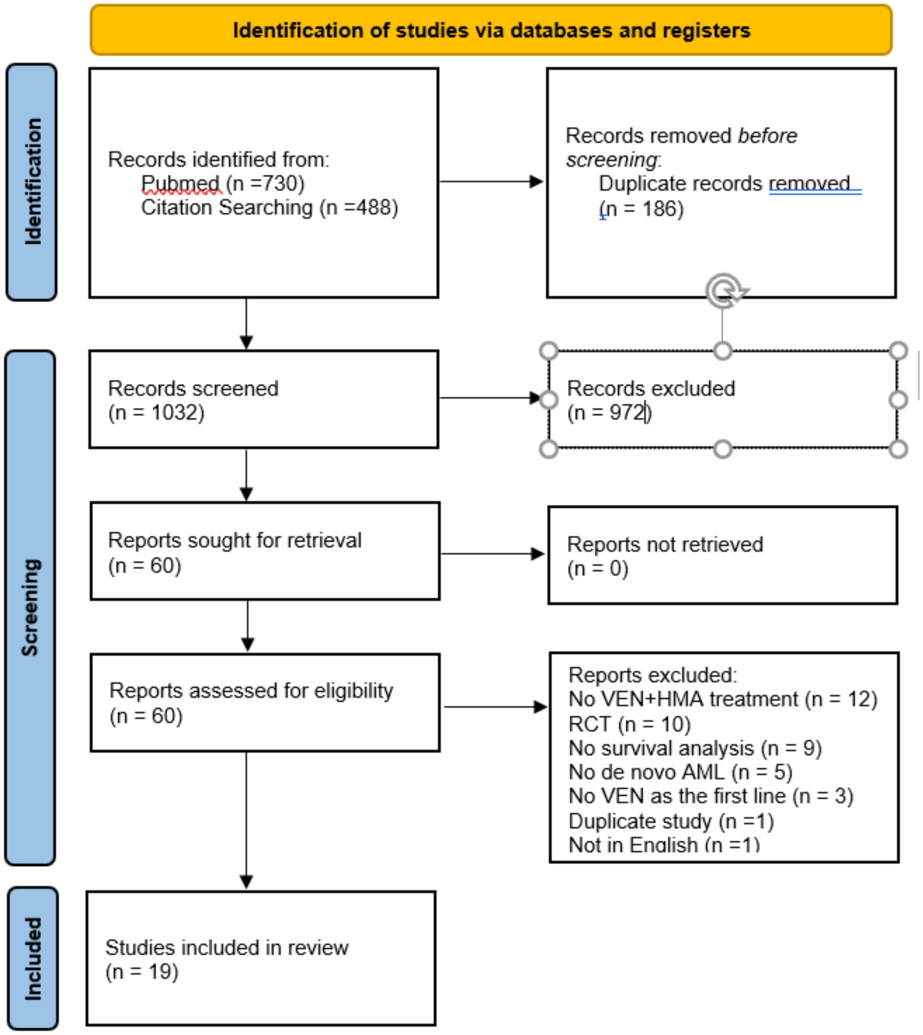

Figure S1. Flowchart.

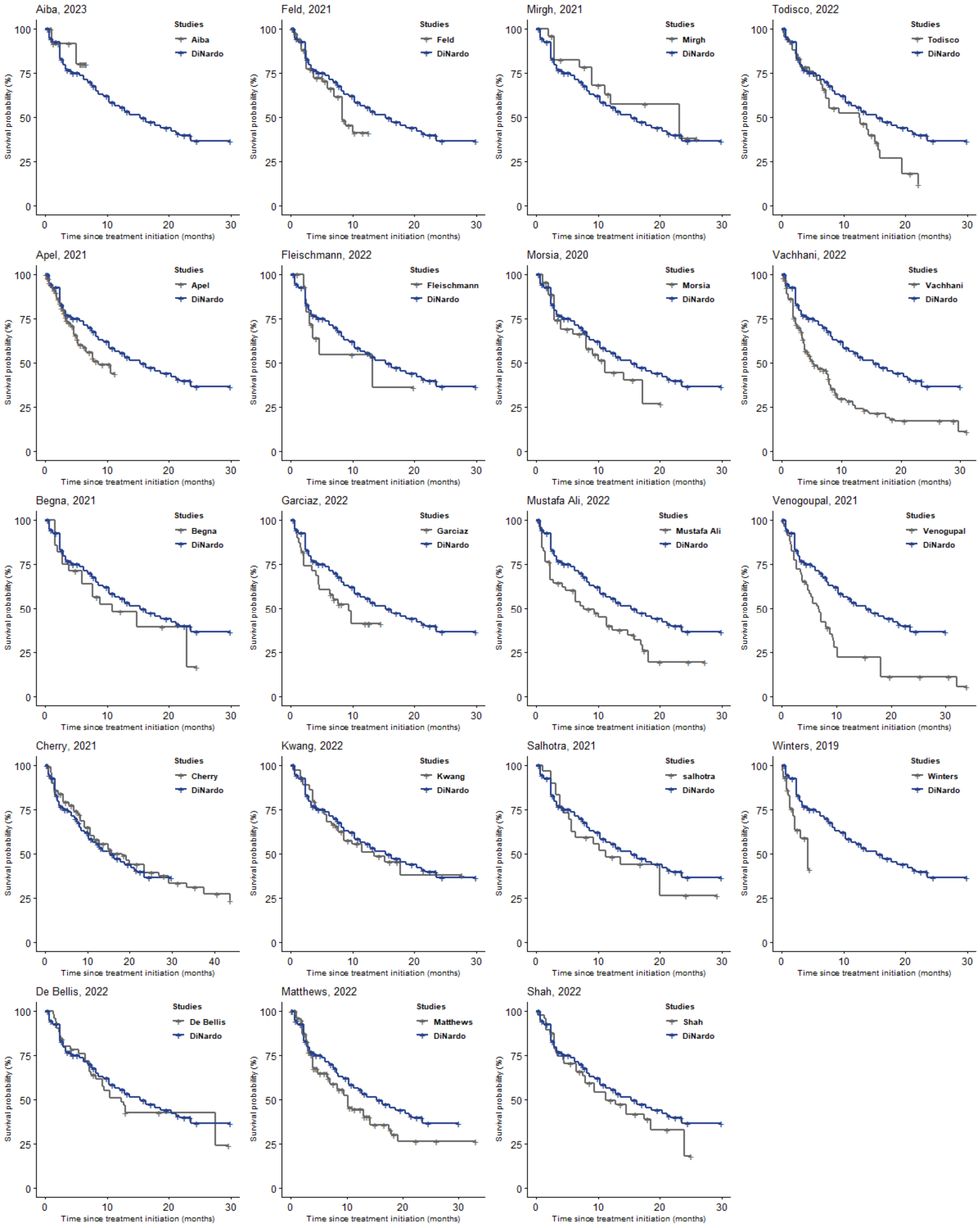

Figure S2. Pairwise comparisons between the study of DiNardo and the real-world studies.

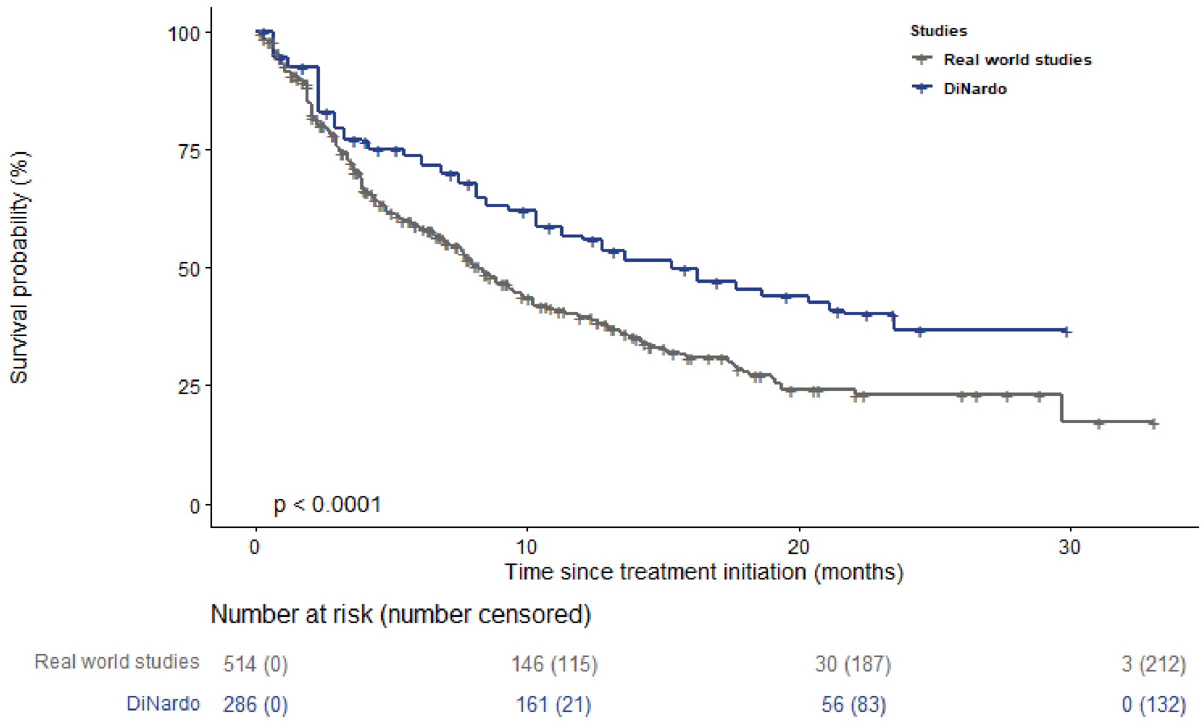

* the studies of Sahah et al., Salhotra et al. and Venogupal et al. were excluded from this analysis

Figure S3. Pooled survival curve after the exclusion of high-risk patients *, compared with that of DiNardo et al.

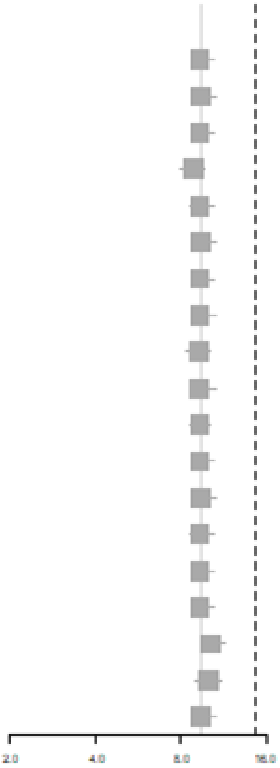

Excluded study	Median survival time (months)	95%CI
Aiba	9.35	(8.78-10.48)
Apel	9.48	(8.87-10.71)
Begna	9.35	(8.78-10.48)
Cherry	8.87	(8.03- 9.79)
De Bellis	9.35	(8.63-10.55)
Feld	9.43	(8.85-10.71)
Fleischmann	9.35	(8.78-10.55)
Garciaz	9.35	(8.81-10.71)
Kwang	9.34	(8.36-10.23)
Matthews	9.34	(8.63-10.71)
Mirgh	9.35	(8.63-10.23)
Morsia	9.35	(8.78-10.48)
Mustafa	9.43	(8.81-10.71)
Sahah	9.35	(8.63-10.48)
Salhotra	9.35	(8.78-10.55)
Todisco	9.35	(8.78-10.48)
Vachhani	10.23	(9.43-11.50)
Venogupal	10.00	(9.04-11.08)
Winters	9.48	(8.87-10.71)

Median survival time - All studies (9.37 months)

Median survival time - DiNardo et al. (14.70 months)

Figure S4. Leave-one-out analysis.
